# Pentoxifylline/Chitosan Films on Wound Healing: In Vitro/In Vivo Evaluation

**DOI:** 10.3390/pharmaceutics15041122

**Published:** 2023-03-31

**Authors:** Vandiara Martins Moreira, Joandra Maísa da Silva Leite, Kaline de Araújo Medeiros, Karoll Moangella Andrade de Assis, Joyce Cordeiro Borges, Lucas Matheus Barreto Santana, Lívia Maria Coelho de Carvalho Moreira, Larissa Pereira Alves, Tharcia Kiara Beserra de Oliveira, João Walter de Souza da Silveira, Dayanne Tomaz Casimiro da Silva, Bolívar Ponciano Goulart de Lima Damasceno

**Affiliations:** 1 Graduate Program fo Pharmaceutical Science (PPGCF), State University of Paraíba (UEPB), Campina Grande 58429-500, PB, Brazil; vandiara.mm@gmail.com (V.M.M.); joandramaisa@hotmail.com (J.M.d.S.L.); karoll.assis@ebserh.gov.br (K.M.A.d.A.); joyce.cborges@ufpe.br (J.C.B.); lucas96barreto@gmail.com (L.M.B.S.); livia.mmoreira@ufpe.br (L.M.C.d.C.M.); larissa.pereira@ufpe.br (L.P.A.); jwsilveira@gmail.com (J.W.d.S.d.S.); dayannecasimiro@servidor.uepb.edu.br (D.T.C.d.S.); 2 Laboratory of Development and Characterization of Pharmaceutical Products (LDCPF), Department of Pharmacy, UEPB, Campina Grande 58429-500, PB, Brazil; 3 Medical Sciences Faculty, University Center UniFacisa, Campina Grande 58411-020, PB, Brazil; tharcia_kiara@hotmail.com

**Keywords:** pentoxifylline, films, chitosan, wound healing, dressings

## Abstract

This study aimed to develop films of chitosan (CSF) associated with pentoxifylline (PTX) for healing cutaneous wounds. These films were prepared at two concentrations, F1 (2.0 mg/mL) and F2 (4.0 mg/mL), and the interactions between the materials, structural characteristics, in vitro release, and morphometric aspects of skin wounds in vivo were evaluated. The formation of the CSF film with acetic acid modifies the polymeric structure, and the PTX demonstrates interaction with the CSF, in a semi-crystalline structure, for all concentrations. The release for all films was proportional to the concentration, with two phases: a fast one of ≤2 h and a slow one of >2 h, releasing 82.72 and 88.46% of the drug after 72 h, being governed by the Fickian diffusion mechanism. The wounds of the mice demonstrate a reduction of up to 60% in the area on day 2 for F2 when compared to CSF, F1, and positive control, and this characteristic of faster healing speed for F2 continues until the ninth day with wound reduction of 85%, 82%, and 90% for CSF, F1, and F2, respectively. Therefore, the combination of CSF and PTX is effective in their formation and incorporation, demonstrating that a higher concentration of PTX accelerates skin-wound reduction.

## 1. Introduction

Wounds can place a significant burden on healthcare systems and are estimated to cost over USD 25 billion annually worldwide to treat chronic wounds [1]. In addition, more than 180,000 deaths occur each year from burns, of which 75% involve bacterial infection in the region [2,3].

The definition of treatment is based on the assessment of multiple variables, such as the depth, extent, and location of the burn; the age of the patient; and trauma associated with diseases. Additionally, most clinical treatments use antimicrobial agents [4]. It is important to highlight that, in the wound environment, there is a loss of function of the innate barrier of the skin and the dermal appendages, which facilitates microbial colonization, since microbes find optimal conditions (temperature, humidity, and nutrients) for their proliferation, which is one of the reasons for the failure to treat patients in wound management, as microbial colonization hinders healing, extends the duration of the inflammatory response, and worsens tissue damage [2,5].

Thus, formulations that protect the wound from external contaminants, facilitate the healing process, reduce the population of microorganisms present over time, remove excess exudate, do not present toxicity and allergic reaction, allow oxygen exchange, and are biodegradable must be designed to reduce the damage caused to the patient and increase the efficiency of tissue recovery [6,7,8,9,10]. Although traditional wound healing formulations including cotton tissue, gauzes, and surgical sutures are widely used in clinical practice, there are disadvantages such as low adhesion and the need for foreign objects for fixation around the injured area [11]. Recently, attention has focused on the development of dressings with biopolymers and bioactive compounds in new pharmaceutical forms such as hydrogel [12], scaffold [13,14], fiber [15], sponge [16], membrane [17], or films [18].

The most used biopolymers for the development of these materials have been alginate [19], PEG [20], PLGA [21], PGA [22], PCL [13], hyaluronic acid [23], and chitosan [13,23,24,25]. The latter is a natural cationic polysaccharide derived from chitin, which consists of N-acetyl-D-glucosamine and D-glucosamine units. Several studies have demonstrated its biocompatibility, biodegradability, potential for modification and personalization (microparticles, nanoparticles, and films), and antibacterial activity. It is from this perspective that chitosan films can overcome the limitations of traditional dressings used in healing, as they can provide adequate protection against bacterial infections, promote a humid environment, and facilitate the regeneration of new tissues, providing a scaffold for cell growth and proliferation [26,27,28,29]. Additionally, it acts as a wound healing accelerator, being beneficial for each phase of healing; however, new alternatives that demonstrate an increase in the aspect of action and act synergistically with chitosan should be studied [30].

Several experimental investigations with drugs and other therapeutic modalities have been used to mitigate free radical processes, stimulate tissue repair, decrease tissue edema, and increase the quality of life of patients. Pentoxifylline (PTX), a xanthine-derived peripheral vasodilator drug, is administered orally or parenterally for circulatory disorders and occlusive peripheral arterial disease [31]. In addition, some studies report that this drug can affect some cytokines such as tumor necrosis factor-α (TNF-α), IL-1, IL-6, IL-8, VEGF, and TGF-β1. These affect the quantitative expression of extracellular matrix metalloproteinases responsible for tissue repair and remodeling in response to injury [32]. Few studies suggest that the topical administration of PTX has considerable potential in the treatment of skin wounds [33,34].

Given this, the design of low-cost materials that are more convenient to use and promote faster healing is important, and, therefore, the objective of this study was to develop chitosan films associated with pentoxifylline for the healing of skin wounds. 

## 2. Materials and Methods

Pentoxifylline (PTX) was kindly donated by Fagron (São Paulo, SP, Brazil) with 99% purity. Chitosan (CS) was purchased from Sigma Aldrich, St. Louis, MO, USA (degree of deacetylation > 85%, Mw = 30,000), and was used as a film-forming polymer. Other solvents such as glacial acetic acid Labsynth (São Paulo, SP, Brazil), ethanol Neon (São Paulo, SP, Brazil), and purified water, which was prepared using an ultrapure water system, were used. All chemical solvents were of analytical grade and were used without purification.

### 2.1. Film Preparation

Chitosan films (CSF) and films containing the drug PTX (PTXCSF) were prepared under different concentrations using the solvent evaporation method (casting). For chitosan films (CSF), a 1% (*w*/*v*) solution was prepared by dissolving it in acetic acid 1% (*v*/*v*). This solution was subjected to magnetic stirring for 24 h without interruption in the stirring time. After this period, it was vacuum filtered (qualitative filter paper 125 mm with retention of particles greater than 12 µm) to remove any insoluble material, thus obtaining a polymeric solution of CS. Then, the solution was poured into a Petri dish (diameter of 5.5 cm, in a volume of 5 mL) and subjected to drying for solvent evaporation in an air circulation oven (TE-394/2 TECNAL Piracicaba, São Paulo, Brazil), at a constant temperature of 50 °C for 24 h. The PTXCSF was prepared by solubilizing PTX in water. Thus, 1 mL of the PTX solution was added to the CS solution, obtaining final concentrations of 2 mg/mL (F1) and 4 mg/mL (F2). These solutions were continuously stirred for 24 h and then filtered and poured into a Petri dish and dried under the same conditions described above. For all systems, photomacrographs were obtained with a digital camera (Nikon^®^ D5300 24.2 MP + Tamron^®^ lens 16–300 mm F/3.5–6.3 Di II VC PZD MACRO).

### 2.2. Characterization of Films

All analyzes were performed in triplicate for the correct characterization of the films produced for this research. Analyzes of CS, PTX, CSF, F1, and F2 were performed.

#### 2.2.1. Scanning Electron Microscopy (SEM)

The photomicrographs of the PTX, CSF, F1, and F2 samples were observed with a TESCAN VEGA 3 microscope (Tescan Analytics, Fuveau, France) using secondary electron images at 2.0 kV for PTX and 8.0 kV for the film formulations increased on a 20 µm scale to examine their surface morphology and internal structure. The sample was mounted with platinum several micrometers thick, which was deposited on the surface of the sample to prevent loading and protect the bottom surface from being damaged by the ion beam.

#### 2.2.2. Fourier Transform Infrared Spectroscopy (FTIR)

Fourier transform mid-infrared spectroscopy analysis was performed using the Perkin Elmer^®^ Spectrum 400 FT-IR/FT-NIR Spectrometer equipment (Waltham, MA, USA) using a universal attenuated reflectance accessory (UATR-FTIR) with crystal in its diamond upper base and a zinc selenide focusing element. The analyzed samples were performed with a scan from 4000 to 600 cm^−1^ with a resolution of 4 cm^−1^.

#### 2.2.3. X-ray Diffraction (XRD)

The diffractograms were obtained using a Shimadzu diffractometer (model XRD 6000, Tokyo, TYO, Japan) equipped with a copper anode. The samples were prepared under a glass support with a thin layer of powder material and were analyzed with 2–45° angular scanning at a scanning speed of 0.5° min^−1^, using Cu radiation (k_α1_).

#### 2.2.4. Differential Scanning Calorimetry (DSC)

Differential scanning calorimetry (DSC) curves were obtained on a DSC Q20 (TA Instruments, New Castle, DE, USA). The samples (2000 ± 0.005 mg) were placed in hermetically sealed aluminum crucibles. The analysis was performed under the following conditions: from 25 to 335 °C, at a heating rate of 10 °C/min, in a nitrogen atmosphere with a flow of 20 mL/min.

### 2.3. In Vitro Release Test with Franz Cells in Synthetic Membrane

The in vitro release study of the prepared F1 and F2 films was evaluated in Franz-type vertical diffusion cells, with a diffusion area of 0.7539 cm^2^, with receptor chambers of an approximate volume of 7 mL, using artificial hydrophilic acetate membranes of cellulose with a pore diameter of 0.45 µm (Millipore, Barueri, Brazil). The receptor compartment was filled with a 7.4 phosphate buffer saline solution (PSB) and ethanol cosolvent in a ratio of 60:40, in a system composed of six individual cells connected to a thermostated bath at 37 ± 0.5 °C under constant stirring at 100 rpm with a magnetic stirrer for a period of 72 h. Acetate membranes were placed on top of the recipient cell and in the donor compartment under this membrane, where the F1 and F2 films were directly arranged, being adjusted to the diffusional area, resulting in a real PTX concentration of 1.37 and 2.74 mg for F1 and F2, respectively, and being after the closed system.

Receptor solution samples were collected at predetermined times of 0.25, 0.5, 1, 1.5, 2, 4, 6, 8, 12, 18, 24, 30, 36, 42, 48, and 72 h. All the solution in the receiving compartment was collected and immediately replaced with PBS to maintain the system’s sink conditions. The cumulative amount of PTX released through the membrane was calculated taking into account the area (µg/cm^2^), with results plotted as a function of time. The values were quantified in a UV-Vis spectrophotometer (SHIMADZU 1800, Kyoto, Japan) (λ = 273 nm).

PTX release kinetics were evaluated using five different theoretical mathematical models using in vitro transdermal drug release data (Shah, 2017); these were the zero-order (µg/cm^2^ versus time), first-order (log µg/cm^2^ versus time), Higuchi (µg/cm^2^ versus time), Korsmeyer–Peppas (log µg/cm^2^ versus log time), and Hixson–Crowell models. It was verified which model best described the release of the drug from the films, considering the value of the correlation coefficient (r), using the equations (Equations (1)–(4)) described:

- Zero-order [35]

(1) $$Q=Q_{0}+k_{0}t$$

- First-order [36]

(2) $$logC_{t}=logC_{0}-k_{t}/2.303$$

- Higuchi Model [36]

(3) $$Q=k_{H}t^{\frac{1}{2}}$$

- Korsmeyer–Peppas Model [37]

(4) $$\frac{Mt}{M\infty }=kt^{n}$$

- Hixson–Crowell Model [38]

(5) $$C_{0}^{1/3}-C^{n}=kt$$

where *Q* is the amount of drug released, *Q*_0_ is the initial amount of drug in solution, *k*_0_ is the zero-order release constant, *C*_0_ is the initial drug concentration, and *C_t_* is the drug concentration in solution at time *t*. *M_t_*/*M*∞ is the fraction of the drug released at time *t*, and *k* is the rate constant.

### 2.4. Study of Wound Healing In Vivo

#### 2.4.1. Animals

To evaluate the wound healing efficiency of the films, the F1 and F2 formulations were used in male and female Swiss mice weighing between 25 and 35 g. The animals were housed in polypropylene boxes suitable for rodents and kept at a temperature of 22 ± 2 °C and relative humidity of about 60 ± 15%, with a light/dark cycle of 12 h, and fed with standard laboratory chow and water *ad libitum*. All experiments were conducted following the protocols approved by the Ethics Committee for the Use of Animals (ECUA) of the Faculty of Medical Sciences of Campina Grande/PB (FCM/CESED) (Protocol number: 0076022022018).

#### 2.4.2. Wound Healing Assay: Treatment Groups, Clinical and Morphometric Analysis

To perform the experimental skin wounds, the animals were anesthetized by an intraperitoneal injection containing ketamine 100 mg.kg^−1^ and xylazine 0.05 mg.kg^−1^. The dorsal region was shaved and two 7 mm diameter circular skin excisions were later performed with the aid of a dermatological punch, from which the skin fragments were removed, leaving the *panniculus carnosus* exposed. [39,40,41,42].

The treatment groups are described in Table 1. Topical treatments were applied daily (once a day) from the second day after a skin-wound formation (the day of experimental wound formation was considered D_0_) until the penultimate day of the experiment. On days 2, 4, 6, and 9, the wounds were photographed to aid clinical assessment and perform a morphometric assessment.

To evaluate the evolution of the clinical aspects of wound healing, a macroscopic analysis was performed, observing these phlogistic signs: edema, hyperemia, and formation of crust and exudate, on days 2, 4, 6, and 9 after the surgical wounds were performed [43]. In addition, the macroscopic efficacy of the treatments was evaluated by determining the percentage of wound closure throughout the experiment. Therefore, on days 2, 4, 6, and 9 of the study, the wounds were photographed using a digital camera (Nikon^®^ D5300 24.2 MP + Tamron^®^ lens 16–300 mm F/3.5–6.3 Di II VC PZD MACRO) at a fixed distance of 15 cm from the animal. The images of the wounds, in high definition, were obtained together with the image of a line on a graduated scale kept next to the wound that allowed the measurement of the wound areas (mm^2^) using the ImageJ^®^ software (National Institutes of Health, Bethesda, MD, USA) [44,45,46]. The residual area of each wound was calculated using Equation (6):

(6) $$Residualwoundarea\left(\%\right)=\frac{Currentwoundarea\left(px^{2}\right)}{Initialwoundarea\left(px^{2}\right)}\times 100$$

### 2.5. Statistical Analysis

Data were expressed as mean ± standard deviation (SD). One-way analysis of variance (ANOVA) followed by the *post hoc* Neuman–Keuls test was used for comparison between groups. Differences with * p < 0.05 were considered significant.

## 3. Results and Discussion

### 3.1. Film Development and Characterization

**Figure 1:**
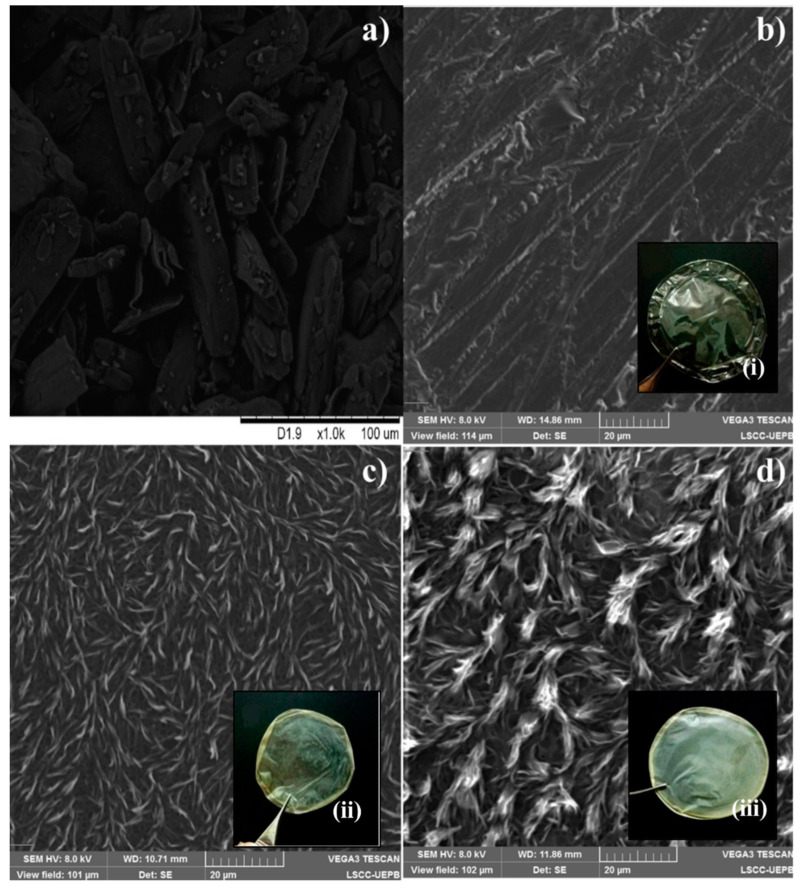
Surface morphology of PTX (pentoxifylline) (**a**), CSF (chitosan films) (**b**), F1 (2.0 mg/mL of pentoxifylline) (**c**), F2 (4.0 mg/mL of pentoxifylline) (**d**) at 20 µm scale. Photomacrographs CSF (**i**), F1 (**ii**), and F2 (**iii**).

The study of the morphology and characteristics of the surface of the films is represented in Figure 1, with photomicrographs analyzed by SEM and photomacrographs. The PTX powder (Figure 1a) has an acicular shape with irregular crystal sizes and polydisperse size distribution. The shapes have a jagged edge structure and are not sticking together. The CSF (Figure 1b) presents itself as a matrix with a homogeneous and non-porous surface with irregularly shaped folds [47,48]. Macroscopically, it presents a completely translucent color with flexible and elastic characteristics (Figure 1b(i)).

On the other hand, films F1 and F2 (Figure 1c,d) showed surface modification when PTX was incorporated into the system and modification in the distribution in the polymer matrix, with no irregular crystal sizes being observed, explained by the amorphization of the drug. In F2 the degree of irregularity was higher when compared to F1, due to the higher concentration of PTX in this film altering the surface morphology of the films. This becomes evident in the photomacrographs in Figure 1c(ii),d(iii), where they exhibit a slightly yellowish coloration and apparent turbidity, due to the incorporation of the drug in the polymeric matrix.

#### 3.1.1. FTIR Spectroscopy Analysis

**Figure 2:**
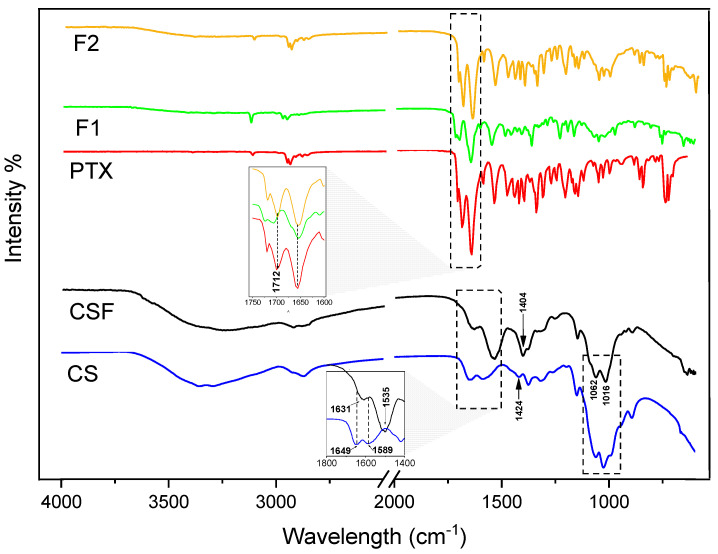
Fourier transform infrared spectroscopy of CS (chitosan, blue line), CSF (chitosan films, black line), PTX (pentoxifylline, red line), F1 (2 mg/mL of pentoxifylline, green line), and F2 (4 mg/mL of pentoxifylline, golden line) spectra.

The analyses of possible interactions that may exist between the materials were observed using FTIR and the spectra are shown in Figure 2. The CS showed a broad absorption band of 3352 cm^−1^ attributed to the elongation of the hydroxyl groups (O-H) and a C-H axial stretch band at 2872 cm^−1^, and at 1649 cm^−1^ a double bond deformation band was observed (C=O) of amide I, and a symmetrical plane of angular deformation of amide II was observed at 1589 cm^−1^; finally, at 1062–1025 cm^−1^, specific bands of the C-O and C-O-C glycosidic bond were observed [49,50,51,52].

As for CSF formed from acetic acid as solvent, it was observed that the characteristic band of amide II at 1589 cm^−1^ was shifted to 1535 cm^−1^, increasing the intensity, which suggests the presence of -NH in this film or even at the insertion of new amino groups in the chitosan structure, due to the electrostatic interaction between chitosan and acetic acid, and the reconstruction of a new network of CS hydrogen bonds during film formation, as previously verified by Qiao, et al. and Zhang, et al. [52,53,54,55]. In addition, a band shift at 1404 cm^−1^ is observed related to the vibrations of the carboxylate ion -COO- and an increase in the intensity of the bands at 1062 cm^−1^. In addition, a band shift at 1404 cm^−1^ is observed related to the vibrations of the carboxylate ion -COO- and an increase in the intensity of the bands at 1062 cm^−1^ and a shift at 1016 cm^−1^ which, due to the rearrangement of the network, affects the bonds between the monosaccharides, and a shift at 1016 cm^−1^ which, due to the rearrangement of the network, affects the bonds between the monosaccharides [54,55].

PTX has characteristic bands at 2945 cm^−1^ referring to the CH stretching, at 1700 cm^−1^ and 1658 cm^−1^ referring to the C=O stretching vibration of the amide and the ketone stretching, respectively, 1545 cm^−1^ vibration of amide bending (N-H), at 1354 cm^−1^ amide elongation (C-N), and an intense absorption band in the 753 cm^−1^ range characteristic of aromatic stretching (C-H) bonds in the form of out-of-plane bending [34,56]. The spectra of the films (F1 and F2) showed vibrational bands typical of PTX and overlapped those of chitosan. For the F2 spectrum, there are bands in the same wave numbers as the PTX spectrum, while for F1 there was a reduction in the intensity of the bands, due to the concentration of PTX in the film, as well as the displacement of the main bands, specifically at 1712 cm^−1^, which may demonstrate some interaction of these groups with the polymer.

#### 3.1.2. X-ray Diffraction (XRD)

**Figure 3:**
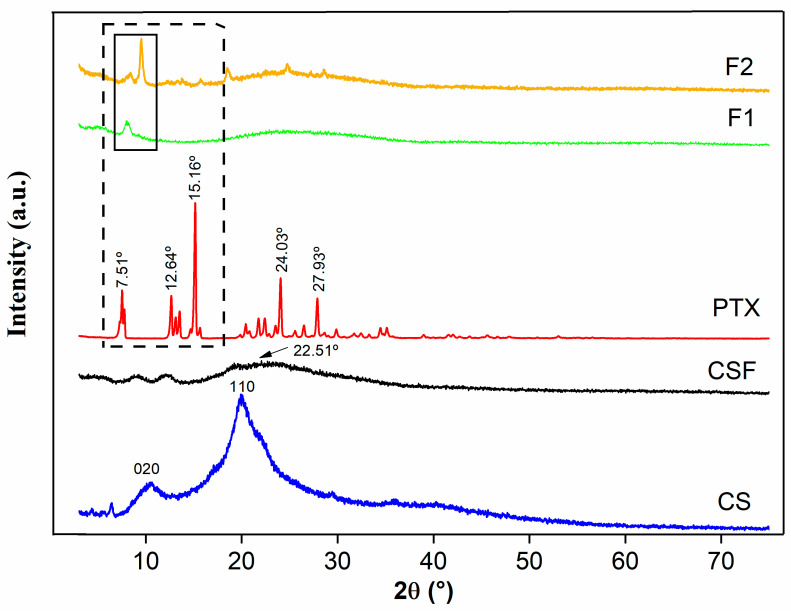
X-ray diffraction spectrum of CS (chitosan, blue line), CSF (chitosan films, black line), PTX (pentoxifylline, red line), F1 (2 mg/mL of pentoxifylline, green line), and F2 (4 mg/mL of pentoxifylline, golden line).

The X-ray diffractograms are shown in Figure 3. For CS, due to the presence of NH_2_ groups in the polymeric chain, which cause the formation of hydrogen bonds between the polymeric chains [57,58], there is the presence of two main diffraction peaks in 2θ of 10.52° in the [020] plane and 19.94° in the (110) plane, demonstrating a semi-crystalline structure, as described in the literature [52,59,60,61]. However, when undergoing solubilization in acetic acid, forming CSF, the degree of crystallinity was reduced with the peak of 19.94° decreasing and shifting to 22.51° and the formation of halos. This indicates an amorphous state, since the interaction of the acid with the NH_2_ bonds can reduce the spaces between the polymer chain and, in a way, participate in the partial rupture of the bond network and, consequently, decrease the crystallinity [52,62].

The same was verified for F1 and F2, which showed reflections of CS in different crystalline planes not being observed as the characteristic reflections of PTX, and this drug confirmed its crystalline structure through the presence of its well-defined reflections at 7.51°, 12.64°, 15.16°, 24.03°, and 27.93° [63], with all of these crystalline reflections being absent in the films, suggesting the dispersion of PTX in the CSF matrix. In addition, the influence of the possible interaction between CS and PTX in this drug dispersion may have influenced a tendency to form organizational plans, as observed in a peak at 8.27° in F1. The same reflection is present in F2, which, due to the increase in concentration, gave rise to another plane at 9.53°, with both planes being of a semi-crystalline structure.

#### 3.1.3. Differential Scanning Calorimetry

**Figure 4:**
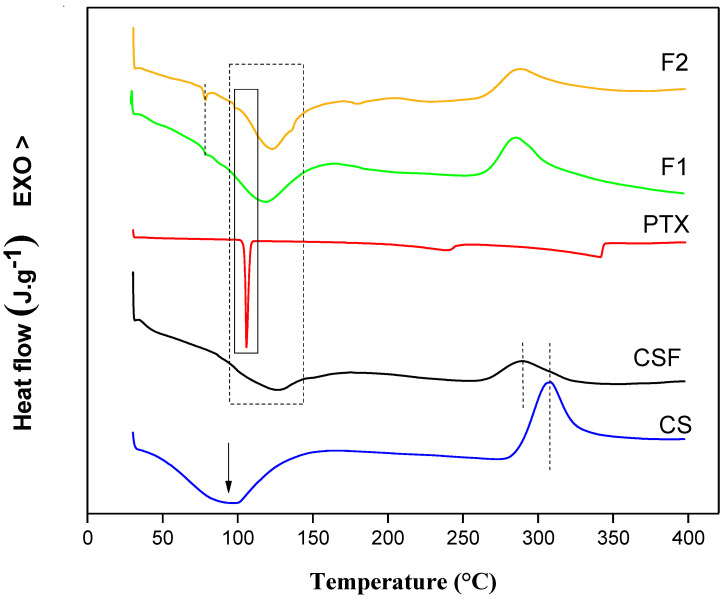
Differential scanning calorimetry thermograms of CS (chitosan, blue line), CSF (chitosan films, black line), PTX (pentoxifylline, red line), F1 (2 mg/mL of pentoxifylline, green line), and F2 (4 mg/mL of pentoxifylline, golden line) components.

The DSC curves are shown in Figure 4. It is possible to observe for CS an endothermic peak T_peak_ = 96.12 °C that is associated with polymer melting and an exothermic peak T_peak_ = 305 °C indicating the thermal decomposition of the polymer chain [52,64,65]. These peaks were shifted to different values when the CSF was prepared in acetic acid, where they obtained values between T_peak_ = 126.05 °C and T_peak_ = 289.01 °C, respectively. These observations indicated that CS and CSF are semi-crystalline, as previously verified, confirmed by the presence of crystalline peaks in X-ray diffraction. Likewise, the films showed values associated with CS melting at T_peak_ = 118.91 °C and 123.93 °C for F1 and F2, respectively. The PTX sample showed an endothermic event at 105.11 °C corresponding to the melting of the drug [66,67,68]. However, in the films, this event was shifted to T_peak_ = 78.04 °C due to the interaction of PTX with CS, showing a peak in the same temperature range for both F1 and F2 films.

### 3.2. In Vitro Release Study

**Figure 5:**
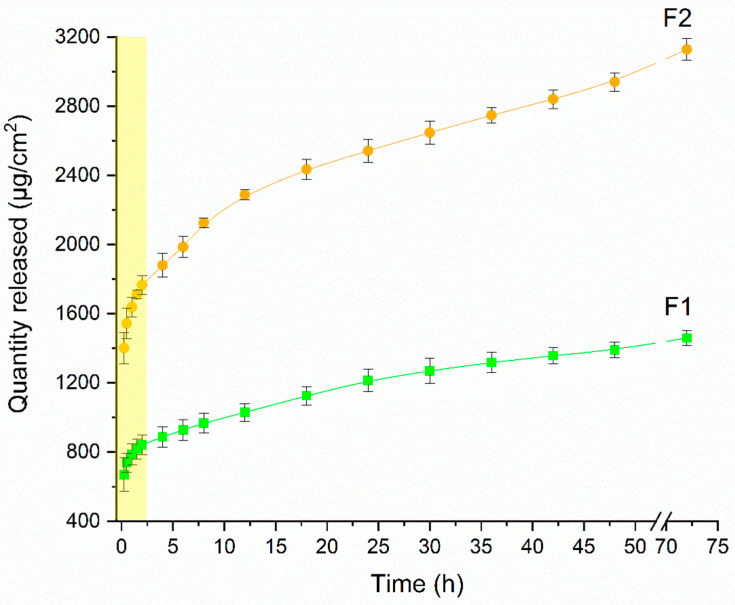
In vitro release profiles in Franz cell apparatus of the drug pentoxifylline incorporated into chitosan films at two concentrations, F1 (1.37 mg of pentoxifylline) and F2 (2.74 mg of pentoxifylline).

The analysis of the release study results is represented in Figure 5 and presents the PTX release profile in the F1 and F2 films. The release of both formulations increased over time, with the first 2 h having a higher speed, with 45.98% and 48.58% released corresponding to 842.83 µg/cm^2^ and 1766.41 µg/cm^2^ for both formulations F1 and F2, respectively. This release may be associated with the amount of amorphous drug present on the surface of the film. After that, the profile demonstrated a controlled release of the drug in the medium due to the swelling of the polymer, in which the drug is released through the mesh to the medium and is influenced by the concentration. In F2, which has a higher concentration, the greater amount of PTX was released during the study period. The maximum concentrations released during the 72 h of the study were 1459.67 and 3128.89 µg/cm^2^ for F1 and F2, respectively. This corresponds to 82.72 and 88.46% of the drug in the F1 and F2 films, respectively. Thus, the developed films showed a release rate proportional to the increase in drug concentration, with the F2 film having a greater amount released when compared to the F1 film, presuming a greater amount of PTX available to exert its action during wound treatment [69].

Five different mathematical models were used to determine the drug release mechanism in vitro with linear regression analysis to select the model that best described the mass transfer phenomena. Table 2 shows the results obtained with adjustment. In both stages (t ≤ 2 h and t ≥ 2 h), the Korsmeyer–Peppas model showed r^2^ > 0.99 for both formulations F1 and F2 in both release stages. According to these data, the demonstrated fit provides an *n* < 0.5 which demonstrates a Fickian diffusion-mediated release of PTX in the polymer matrix. There is a rapid release at the beginning that reduces over time, describing a linear relationship between the square root of time and the cumulative amount of PTX released from the films, suggesting that this release was controlled by a usual molecular diffusion mechanism of the drug. This transfer rate per unit area is proportional to a concentration gradient or chemical potential between the two sides of the diffusion layer [70,71].

### 3.3. In Vivo Study

#### Clinical Aspects and Morphometric Analysis of Skin Wounds

**Figure 6:**
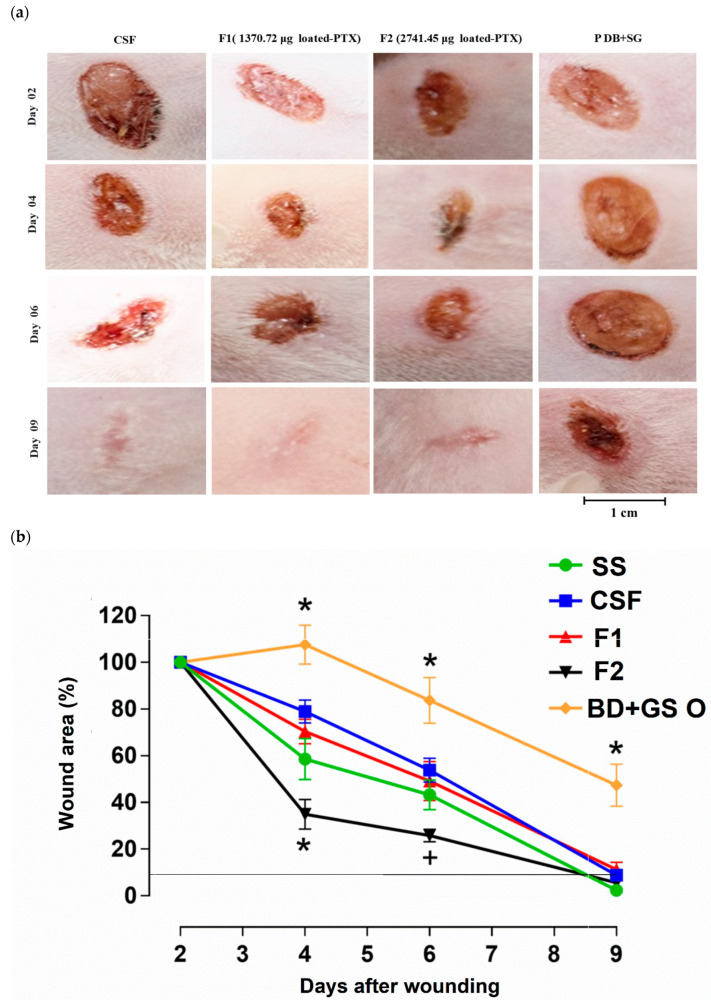
Wound healing process: (**a**) photographs of mechanical wounds of the four groups during the wound healing treatment period at 2, 4, 6 and 9 days; (**b**) percentage of wound area reduction from 2, 4, 6, and 9 days. Data are expressed as mean ± SD (*n* = 7) and * *p* < 0.05 different from all other groups; + *p* < 0.05 different from CSF (chitosan films), F1, BD + GS O groups; one-way ANOVA followed by the Neuman–Keuls test.

The healing potential was established from the daily analysis, which was performed to verify the clinical aspects of the lesions of the different groups (test and control). The wounds were photographed every 2 days (without the films) as shown in Figure 6a,b, which shows the healing process and wound reduction.

Injured mice showed a tendency for wound reduction up to day nine; however, the healing rate and effect were different. At the beginning of treatment (D_0_), all wounds in all groups had a similar appearance (bright red color, reflecting the blood caused by the physical trauma). From the second post-wound day, aspects began to differ between the groups studied. From day 2 to day 4, there was a reduction in the size of the wound without perilesional redness and the appearance of a brown crust with contraction of the edges, which proved to be thicker and stiffer in the groups treated with the films, obtaining individual capacity (percentage) of reduction of up to 60% for F2, a significantly higher value when compared to F1, which reduced by 30%. For CSF, the decrease was only 20%, as seen in Figure 6. It is important to highlight that the occurrence of mild inflammation in the initial phases of healing is beneficial, as it allows the induction of tissue healing [72]. Meanwhile, the positive control group BD + GS O did not show a significant reduction (*p* < 0.05) in wound size until the first 4 days; only after the 6th day was there a 20% reduction in the wound area. On that day, the reduction process for F2 reached 70%, followed by 50 and 40% for F1 and CSF, respectively.

In all groups, a brown crust with greater rigidity can be observed. At the end of the ninth day, F1, F2, and CSF obtained a reduction of 82%, 90%, and 85%, with brown crust detachment, the tissue being pink, undamaged, and healthy. This result differs from the BD + GS O group, which reduced by only 50% with tissue exposure without complete healing and with brown crust and contraction of the edges. Similar observations were found in previous studies in the field of tissue regeneration [72,73,74,75,76,77]. In addition, it is important to highlight that the film, unlike the ointment, has micropores that allow greater circulation of oxygen at the lesion site and greater adherence to the affected site. This allows not only the carrying but also the controlled release of the drug by diffusion, as seen above, in addition to facilitating the removal of exudates present [25].

The CS film with the highest amount of PTX (F2) increased the speed and percentage of wound reduction, due to the dispersion of the drug in the polymeric mesh. PTX has multi-mechanisms of action, it improves blood flow, and it may be responsible for the reduction of neutrophils, free radicals, and proteolytic enzymes that degrade the extracellular matrix, as well as TNF-α [33]. Furthermore, it is responsible for the increase in genes that express the tissue inhibitor of metalloproteinase (TIMP-1) and a reduction in the gene expression of matrix metalloproteinase 1 (MMP-1) and MMP-3, demonstrated in a study based on a wound healing model in normoglycemic rats, who had an incision wound in the back and administration to the experimental group of 25 mg/kg of PTX, twice daily per seven days by systemic route, and were submitted to polymerase chain reaction (PCR) testing [78]. That said, a study by Aghjani et al. [34] evaluated the healing process of wounds treated with PTX in niosomes, where they used mice; incisions in the neck area were made, and the 500 mg of conventional and niosomal formulations were applied twice a day completely covering the wounds. After 12 days of treatment, the PTX group on niosomes had a significantly greater wound reduction, with a wound surface area of only 1%. In this case, the study histopathological evaluation was performed, demonstrating that for this formulation, there was a decrease in the number of inflammatory cells at the wound site, indicating an early onset. Furthermore, the differentiation of keratinocytes was confirmed by the appearance of a layer of keratin above the layers of nucleated epithelial cells, with the formation of granulation tissue below the layer of neoepidermis, being completely differentiated for this formulation when compared to the other groups without PTX, with the fibroblasts fully stretched and aligned.

It is important to emphasize that oxidative stress can damage cells and tissues and comprising healing, and PTX may affect healing, reducing this stress [79]. Some studies demonstrate that PTX reduces oxidative stress by reducing the production of reactive oxygen species (ROS) and increases the activity of antioxidant enzymes in the tissue, such as superoxide dismutase (SOD) and catalase, in addition to modulating the expression of genes involved in oxidative stress and inflammation, which may further contribute to its effects on healing [79,80,81,82,83,84]. This drug, when compared to others such as quinacrine, vitamin C, and vitamin E, demonstrated superior antioxidant activity and was more effective against oxidative stress or more effective in preventing lipid peroxidation, respectively [85,86].

In addition to PTX activity, CS also aids in the healing and antimicrobial process. This polymer assists in the bleeding process by promoting platelet aggregation of erythrocytes and inhibiting the dissolution of fibrin in the hemostatic stage; secondly, it helps eliminate bacteria from the wound, through an antimicrobial activity that interferes with the inflammation phase. Finally, it accelerates the proliferation of the skin, promoting the growth of granulation tissue, and stimulating the proliferation stage [25]. Regarding the antimicrobial activity of CS, there are supposedly two mechanisms of action: the first is through its cationic nature and consists of the interaction between the -NH_3_ groups and negatively charged groups on the surface of the bacterial cell wall, preventing the transmission of vital solutes, reducing cell viability. The second is through the diffusion of CS, which diffuses into the cell nucleus, inhibiting the synthesis of bacteria [87]. Additionally, this activity, when compared to other polymers that have been used to prevent bacterial growth, has been shown to have superior activity, particularly against gram-negative bacteria, and it has even been shown to have antibacterial activity comparable or even superior to silver nanoparticles against bacterial species including E. coli, S. aureus, and P. aeruginosa [88,89,90].

It is necessary to understand the process of epithelialization, quantify neutrophils and lymphocytes to determine the tissue regeneration phase, and determine the angiogenic indices and the state of histological maturation, which is a limitation of this present study. Additionally, it is important to note that the use of films of CS with PTX is not necessarily a “solid alternative” to existing commercial wound healing drugs, as commercial products for this purpose may contain a combination of different active ingredients and their effectiveness may depend on the type of wound and patient characteristics. Factors such as wound size, location, and depth, as well as patient comorbidities and medication use, can affect the appropriateness of this treatment approach.

## 4. Conclusions

The PTX was dispersed in the polymeric mesh of the CS, changing the crystalline state of both materials. The choice of solvent (acetic acid) for the formation of the films provided the formation of a semi-crystalline material; that is, the choice of solvent directly influences the performance of the films formed containing CS (CSF, F1, and F2). The interaction between PTX and CS influences the crystallinity of the material, providing different organizational structures. However, all materials showed a positive effect on wound healing with a minimum of 82% and a maximum of 90% wound reduction after nine days of treatment, and the association with the highest concentration (F2) of PTX showed higher healing speed, due to the synergistic effect of these materials, which benefit the treatment and can act through different healing pathways.

Thus, the association of CS and PTX offers improved healing when they are combined, being easy to apply and providing a protective barrier that can help keep the wound clean and moist, in addition to the fact that CS is a biocompatible polymer and well tolerated by the body; in this case, it is being used in an unprecedented way associated with PTX. However, more information is needed to understand the potential side effects or irritability in humans that the PTX or chitosan may cause when associated. Overall, the films prepared in this study have an innovative and promising potential for the production of wound dressings; however, more research is needed to fully evaluate their efficacy and safety compared to existing commercial wound healing products.

## Figures and Tables

**Table 1:** Description of the study groups (*n* = 7), evaluated in treatment and control groups, used for the wound healing trial lasting 9 days.

Group	Treatment	Number of Animals	Duration of the Treatment
G1	Sterile saline solution 0.9% (200 µL) (SS)	7	9 days
G2	CSF	7	
G3	F1 (1370.72 µg PTX)	7	
G4	F2 (2741.45 µg PTX)	7	
G5	Betamethasone Dipropionate Ointment with Gentamicin Sulfate (BD + GS O)	7	
The total amount of animals		35	

**Table 2:** Linear evaluation of in vitro PTX (pentoxifylline) release mathematical models from CS films.

		Formulations			
Models		F1		F2	
		**k_0_ (µg.min^−1^)**	**r^2^**	**k (µg.min^−1^)**	**r^2^**
Zero-order	*t* ≤ 2 h	30.54	0.9519	32.21	0.9585
	*t* ≥ 2 h	1.75	0.9453	1.86	0.9570
First-order		**k_1_ (min^−1^) × 10^−3^**		**k_1_ (min^−1^) × 10^−3^**	
	*t* ≤ 2 h	0.40	0.9735	0.43	0.9790
	*t* ≥ 2 h	0.04	0.9806	0.05	0.9830
Higuchi		**k_H_ (µg.min^−1/2^)**		**k_H_ (µg.min^−1/2^)**	
	*t* ≤ 2 h	39.86	0.9821	42.03	0.9855
	*t* ≥ 2 h	12.48	0.9857	13.25	0.9832
Korsmeyer–Peppas		**k_kp_(min^−*n*^) × 10^−3^**		**k_H_ (µg.min^−1/2^)**	
	*t* ≤ 2 h *	42.81	**0.9958**	45.13	**0.9965**
	*n* *	**0.2060**		**0.1081**	
	*t* ≥ 2 h **	34.98	**0.9906**	39.07	**0.9974**
	*n* **	**0.1074**		**0.1914**	
Hixson–Crowell		**k_HC_ (µg^1/3^.min^−1^) × 10^−3^**		**k_HC_ (µg^1/3^.min^−1^) × 10^−3^**	
	*t* ≥ 2 h	0.12	0.9678	0.13	0.9737
	*t* ≤ 2 h	0.01	0.9854	0.01	0.9852
Best Fit		Korsmeyer–Peppas		Korsmeyer–Peppas	

* *n* value for time ≤ 2 h; ** *n* value for time ≥ 2 h.

## Data Availability

The data presented in this study are available on request from the corresponding author.
